# The Inception of the "Two Trolley Technique" for Supine Positioning in a Patient With Penetrating Injury to the Back

**DOI:** 10.7759/cureus.24020

**Published:** 2022-04-10

**Authors:** Asish Karthik, Shijin PS, Prabhleen Kaur, Rini Sara Varghese, Aiswarya CH

**Affiliations:** 1 Department of Anesthesiology, Government Medical College, Thrissur, Thrissur, IND

**Keywords:** penetrating spinal injury, trauma, supine position, anesthesia induction, difficult airway, anesthesia

## Abstract

Penetrating trauma to the back causes the anesthesiologist many difficulties in airway management and obtaining central lines due to the inability to position supine. Lateral position intubation for the same has been described earlier but still remains unfamiliar. Here, we describe the case of a stab injury to the back and how we achieved the optimal supine position using the "Two Trolley Technique."

## Introduction

Penetrating injuries are an area of concern in emergency departments worldwide. Before one such patient is taken up for surgery, anesthesiologists face difficulty positioning the patient supine to secure the airway and obtain central venous access. This occurs mainly when the injury is in the midline of the back, with the penetrating object protruding outward. Hence, it becomes difficult to attain a supine position without causing harm to the surrounding vital structures of the mediastinum.

We present the anesthetic management of such a rare case and the inception of the "Two Trolley Technique" for supine positioning, a deviation from intubation guidelines in the lateral position for penetrating trauma to the back.

## Case presentation

**Figure 1:**
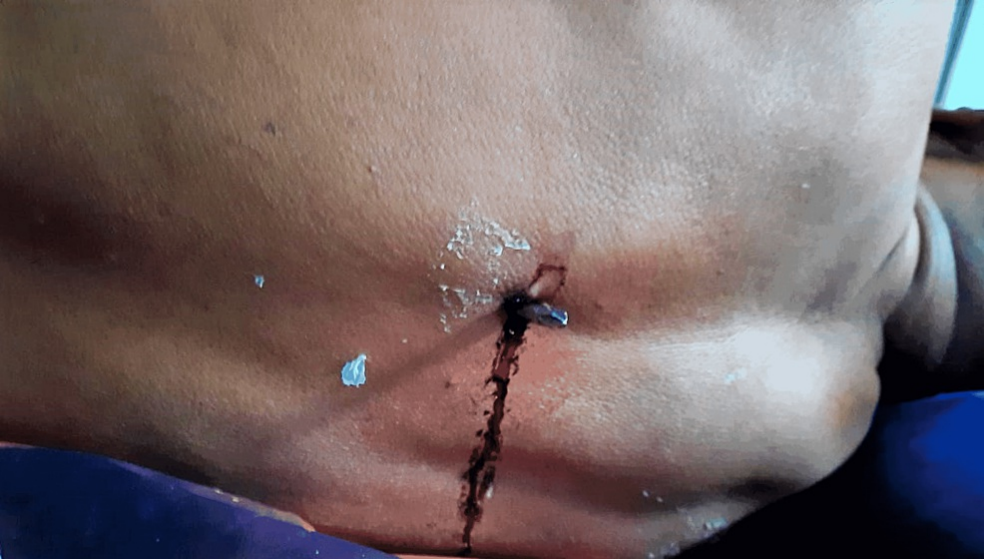
Picture showing the protruding end of the screwdriver.

A 55-year-old male with a history of type II diabetes mellitus and chronic obstructive pulmonary disease was brought to the emergency department with a penetrating injury to the back (Figure 1). He complained of pain at the injured site and inability to move his lower limbs.

**Figure 2:**
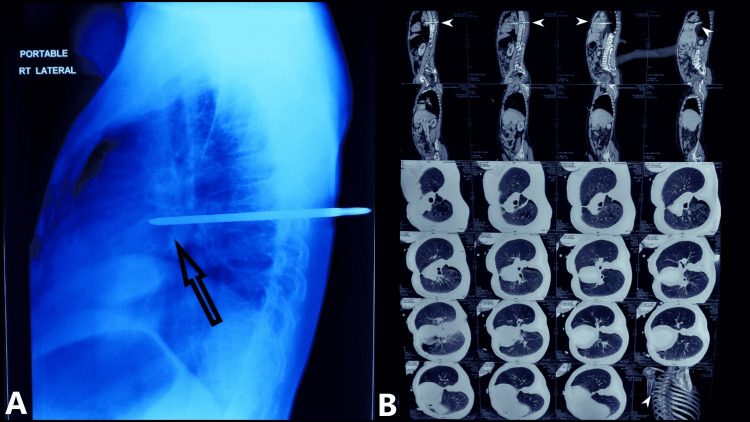
A: Chest X-ray showing the depth of penetration. B: Sagittal CT scan showing the screwdriver abutting the great vessels and transecting the spine as indicated by the white arrowheads.

On examination, an intercostal drainage (ICD) tube was in situ (suspecting lung injury), and he was conscious and oriented with stable vitals, but the wound was oozing blood. The patient had grade 0 power in his lower limbs associated with a bilateral loss of sensation. His airway assessment revealed retrognathism and submandibular fullness. The chest X-ray showed that the depth of penetration was around 80% of the length of the rod as shown in Figure 2A, and the CT imaging showed the screwdriver tip abutting the great vessels of the mediastinum and the pericardium and transecting the spine at the T7-T8 level (Figure 2B).

**Figure 3:**
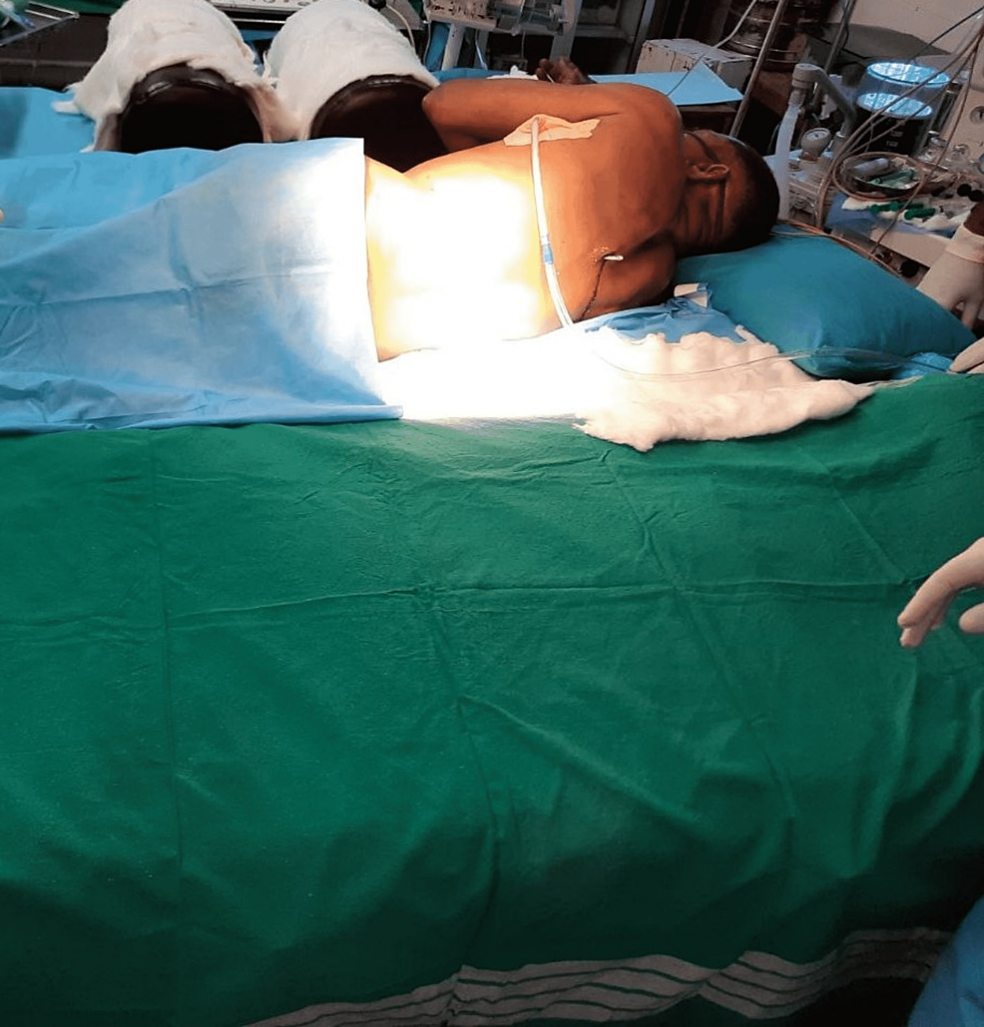
Picture depicting the two approximated trolleys with cotton padding over the gap.

A thoracotomy was planned, and the patient was taken into the premedication room in the lateral position. Plans for strategically positioning the patient supine were made. Two patient trolleys of the same height were used. They were approximated such that a gap of around 5 cm was left between them (Figure 3).

**Figure 4:**
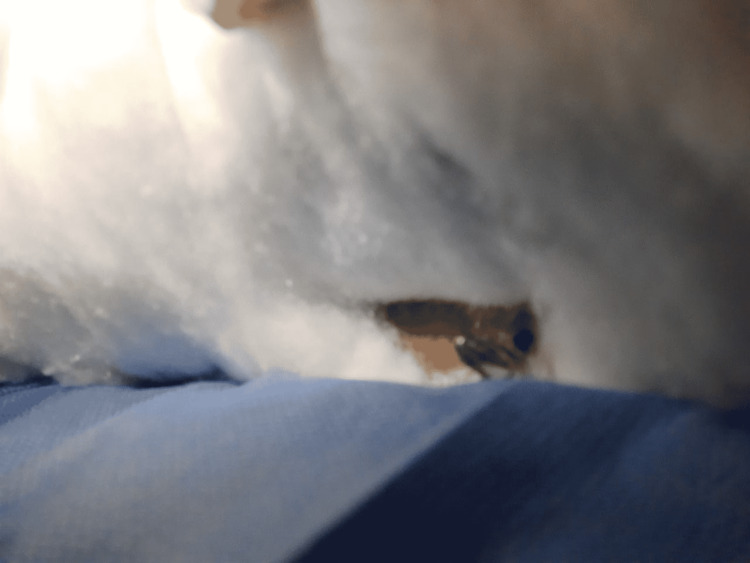
Picture showing the protruding end of the screwdriver positioned in the gap.

It was then padded with cotton, leaving an orifice for the rod to be positioned within (Figure 4).

The wheels were locked, and a wedge-shaped block was kept between them.

**Figure 5:**
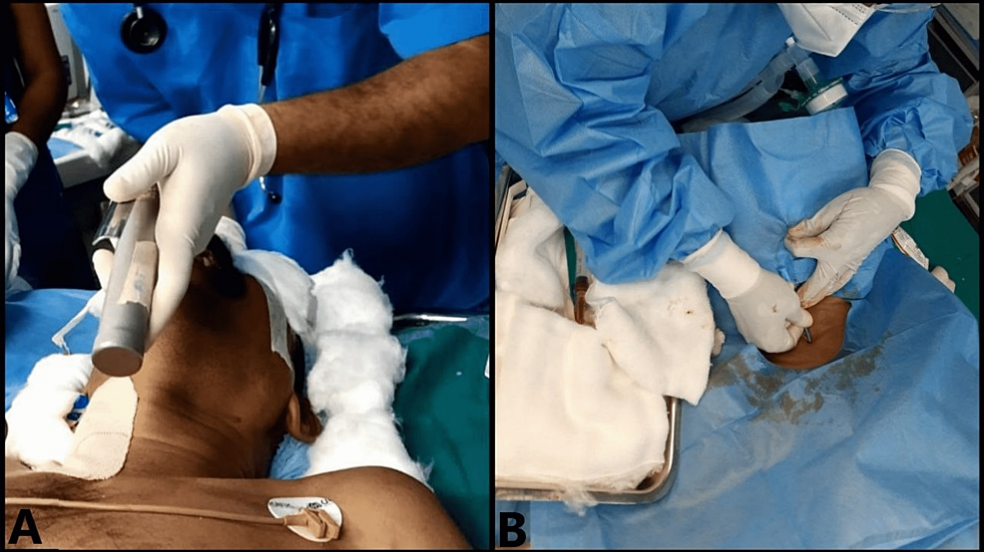
A: Intubation with DLT in supine position. B: Right central venous line obtained. DLT: double-lumen tube

Once the supine position was achieved, the patient was then premedicated, induced, and intubated with a 39 Fr left-sided double-lumen tube (DLT) (Figure 5A), and lung isolation was confirmed. Right internal jugular venous access was obtained using the modified Seldinger technique under ultrasound guidance (Figure 5B).

The patient was then turned to the left lateral position to complete right thoracotomy and lung isolation. The screwdriver tip was found between the major vessels and removed by controlled traction from the outside. The lung laceration was repaired.

A cerebrospinal fluid leak was observed from the spinal canal. The patient was positioned prone, DLT replaced with an 8.5 mm ID flexometallic tube, and the neurosurgeons carried out a laminectomy and closure of the tear. The subsequent intra- and postoperative periods were uneventful.

**Figure 6:**
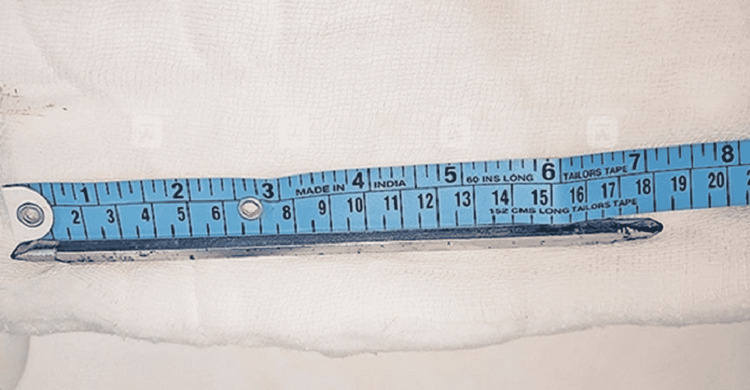
Picture showing the length of the removed rod.

The length of the removed screwdriver rod was 18 cm (Figure 6).

## Discussion

We know that inadequate positioning can result in prolonged or failed intubation and that difficult or compromised airway management is a significant contributor to patient morbidity and mortality [1]. Having a screwdriver penetrating his back and the fear of torrential bleeding, tamponade, or even death in the case of any displacement of the same, along with a difficult airway, this patient posed many challenges for the anesthesiologist.

Achieving the optimal supine position for all the anesthetic interventions was the ultimatum. The available literature showed intubations in the semi-lateral position [2,3] and supine positioning using a surgical trolley and the operation table with a considerable gap between the two for the impaled object [4].

However, our patient was overweight and had a difficult airway. There was a need to place a double-lumen endobronchial tube and no scope of error regarding movement. To overcome these challenges, we came up with the "Two Trolley Technique," with the impaled object positioned perfectly between two patient trolleys. It is an easy technique to attain supine positioning without the fear of any displacement and can be modified for use with or without the cotton padding depending upon the size of the projecting component.

The critical point of concern is to maintain a stable gap so that the penetrating object fits in it. This can easily be achieved by choosing the same kind of trolleys, locking their wheels, and keeping a wedge between them to prevent inward motion.

## Conclusions

The strategic "Two Trolley Technique" can be easily implemented in such cases, with just two similar patient trolleys, which are ubiquitously found in every hospital. It can be compared with lying on two adjacent mattresses, which hardly causes the patient any discomfort. We can restrict the mobility of the penetrating object and, at the same time, not deviate to the uncommon territory of the lateral position intubation.

Hence, we would like to put forward the "Two Trolley Technique" for successful supine positioning of patients with a penetrating injury to the back.
